# First Record of Eocene Bony Fishes and Crocodyliforms from Canada’s Western Arctic

**DOI:** 10.1371/journal.pone.0096079

**Published:** 2014-05-01

**Authors:** Jaelyn J. Eberle, Michael D. Gottfried, J. Howard Hutchison, Christopher A. Brochu

**Affiliations:** 1 University of Colorado Museum of Natural History and Department of Geological Sciences, University of Colorado at Boulder, Boulder, Colorado, United States of America; 2 Department of Geological Sciences and Museum, Michigan State University, East Lansing, Michigan, United States of America; 3 University of California Museum of Paleontology, Berkeley, California, United States of America; 4 Department of Earth and Environmental Sciences, University of Iowa, Iowa City, Iowa, United States of America; University of Pennsylvania, United States of America

## Abstract

**Background:**

Discovery of Eocene non-marine vertebrates, including crocodylians, turtles, bony fishes, and mammals in Canada’s High Arctic was a critical paleontological contribution of the last century because it indicated that this region of the Arctic had been mild, temperate, and ice-free during the early – middle Eocene (∼53–50 Ma), despite being well above the Arctic Circle. To date, these discoveries have been restricted to Canada’s easternmost Arctic – Ellesmere and Axel Heiberg Islands (Nunavut). Although temporally correlative strata crop out over 1,000 km west, on Canada’s westernmost Arctic Island – Banks Island, Northwest Territories – they have been interpreted as predominantly marine. We document the first Eocene bony fish and crocodyliform fossils from Banks Island.

**Principal Findings:**

We describe fossils of bony fishes, including lepisosteid (*Atractosteus*), esocid (pike), and amiid, and a crocodyliform, from lower – middle Eocene strata of the Cyclic Member, Eureka Sound Formation within Aulavik National Park (∼76°N. paleolat.). Palynology suggests the sediments are late early to middle Eocene in age, and likely spanned the Early Eocene Climatic Optimum (EECO).

**Conclusions/Significance:**

These fossils extend the geographic range of Eocene Arctic lepisosteids, esocids, amiids, and crocodyliforms west by approximately 40° of longitude or ∼1100 km. The low diversity bony fish fauna, at least at the family level, is essentially identical on Ellesmere and Banks Islands, suggesting a pan-High Arctic bony fish fauna of relatively basal groups around the margin of the Eocene Arctic Ocean. From a paleoclimatic perspective, presence of a crocodyliform, gar and amiid fishes on northern Banks provides further evidence that mild, year-round temperatures extended across the Canadian Arctic during early – middle Eocene time. Additionally, the Banks Island crocodyliform is consistent with the phylogenetic hypothesis of a Paleogene divergence time between the two extant alligatorid lineages *Alligator mississippiensis* and *A. sinensis*, and high-latitude dispersal across Beringia.

## Introduction

**Figure 1:**
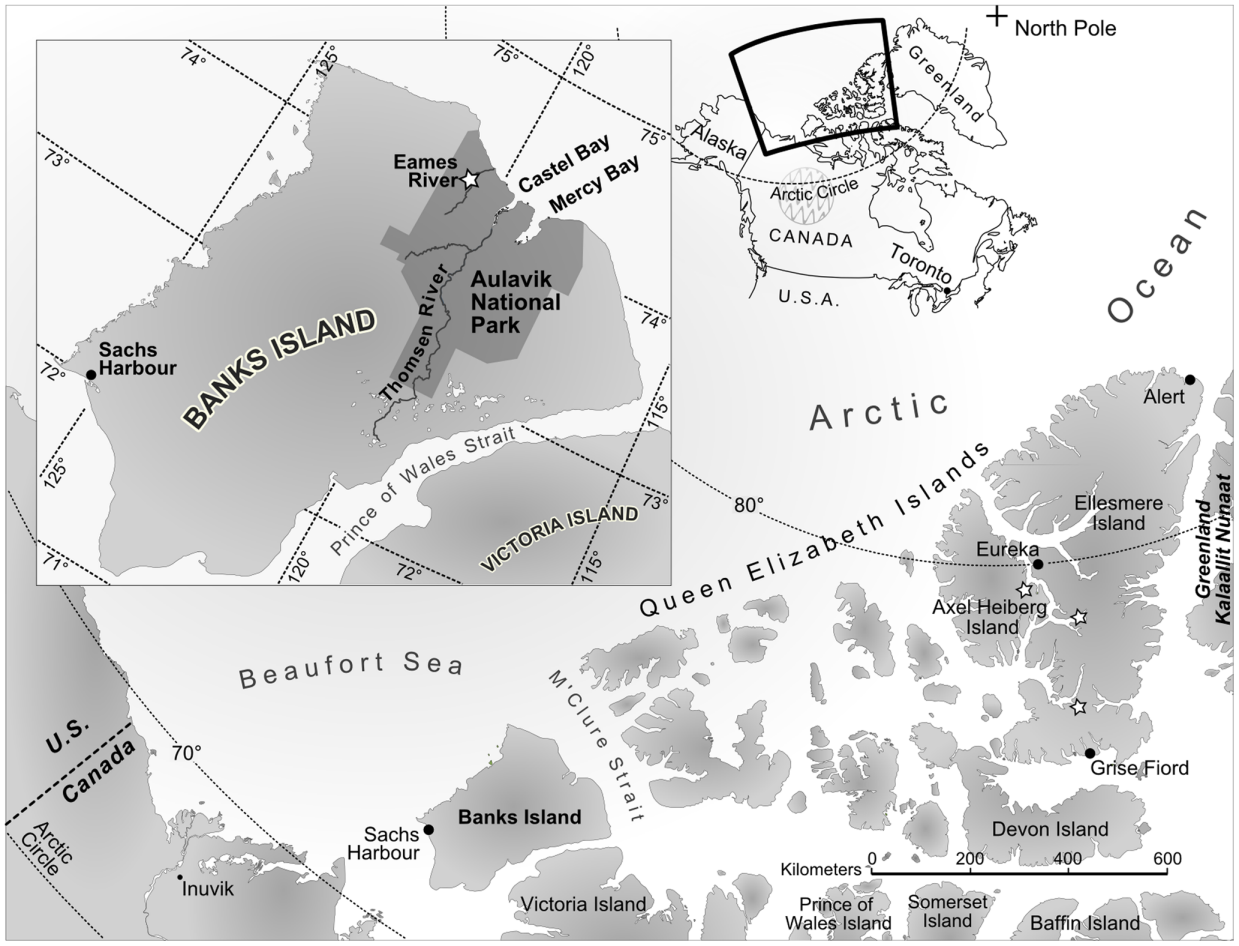
Map of Arctic Canada showing location of Eocene crocodyliform locality on northern Banks Island, NWT (inset). Stars on Ellesmere and Axel Heiberg Islands mark localities from which Eocene crocodylian and bony fish fossils were reported prior to this report [3]. Artwork by L. McConnaughey.

Discovery of Eocene vertebrates, including alligators, turtles, fishes, and mammals, on Ellesmere and Axel Heiberg Islands in Canada’s eastern High Arctic [1], [2], [3] (Figure 1) was a critical paleontological contribution of the last century, as it indicated that this region of the Arctic had been mild, temperate, and ice-free during the early – middle Eocene (∼53–50 Ma), despite its location at ∼76–77°N. paleolatitude [4]. Eocene vertebrate-bearing strata of the Eureka Sound Group crop out on islands across the Canadian Arctic; however, to date, discoveries of Eocene non-marine vertebrates are limited to Ellesmere and Axel Heiberg Islands. On Banks Island – Canada’s westernmost Arctic Island – Eureka Sound strata are exposed extensively across northwestern parts of the island [5], but the paleoenvironment is interpreted as predominantly shallow marine, based upon abundant shark teeth [6], the trace fossil *Ophiomorpha*, marine microfossils, and the sedimentology [5]. Here, we describe the first Eocene non-marine vertebrates from northern Banks Island. Rare fossils of bony fishes, including the lepisosteid (gar) *Atractosteus,* an esocid (pike), and an amiid (bowfin), as well as a single vertebra of a crocodyliform were discovered in lower – middle Eocene strata of the Cyclic Member, Eureka Sound Formation near Eames River within Aulavik National Park (∼76°N. paleolatitude). These fossils extend the known geographic ranges of Eocene Arctic lepisosteids, esocids, amiids, and crocodyliforms west by ∼40° of longitude or ∼1100 km [7]. Additionally, they provide a glimpse into the early – middle Eocene vertebrate fauna from Canada’s western Arctic, hitherto known only from isolated sharks’ teeth [6]. The shark fauna is being described elsewhere.

To date, the majority of paleoclimatic data for the Eocene Arctic has come from eastern Arctic localities [8] and a single locality on Lomonosov Ridge in the central Arctic Ocean [9], [10]. By analogy with living crocodylians [11], the occurrence of a crocodyliform fossil on northern Banks implies that a regionally mild, temperate climate extended across the Canadian Arctic during early – middle Eocene time. Further paleoenvironmental, biogeographic, and phylogenetic implications are discussed below.

## Geologic Setting and Age

The fossils were recovered alongside hundreds of shark teeth from CMN localities BKS04-16 and BKS04-19 in Eocene strata near Eames River, within the boundaries of Aulavik National Park on northern Banks Island, NWT, Canada (∼N 74° 10′; W 120° 45–46′; Fig. 1). Because the locality is within the boundaries of a national park, we are not able to provide more precise coordinates in this paper. Qualified researchers should contact the Canadian Museum of Nature (CMN) in Ottawa, ON, Canada, to request the exact coordinates.

The Eocene bony fish and crocodyliform localities on northern Banks occur in strata initially mapped as the Cyclic Member of the Eureka Sound Formation [5], [12]. Subsequently, these strata were re-assigned to the Margaret Formation of the Eureka Sound Group, and inferred to be correlative with Eocene, terrestrial vertebrate-bearing strata of the Margaret Formation on Ellesmere and Axel Heiberg Islands over 1,000 km away in Canada’s eastern High Arctic [13], [14]. However, given the enormous distance from the type section of the Margaret Formation (at Strand Fiord on southern Axel Heiberg Island) [13], the variable lithology of the Eureka Sound Group across the Arctic, and deposition in multiple isolated basins separated by upwarps [13], the Eocene vertebrate-bearing sediments in Banks Basin on northern Banks Island are here left as the Cyclic Member. There are environmental differences that are consistent with taking this approach, namely that the Margaret Formation in the eastern Arctic is predominantly non-marine, producing palynomorphs and a terrestrial vertebrate fauna, while the Cyclic Member on northern Banks Island comprises coarsening-upward cycles of shale, silt, unconsolidated sand, paleosol, and lignitic coal that are interpreted as a deltaic sequence in a marginal marine setting [5]. At multiple localities, the Cyclic Member preserves abundant shark teeth, bivalves, and the trace fossil *Ophiomorpha*, interpreted as the burrow of a thalassinidean shrimp and generally indicative of shallow-water, moderately high energy, coastal marine environments [15]. Marine microfossils (foraminiferans and radiolarians) also are documented from the Cyclic Member [5].

The bony fish and crocodyliform fossils were recovered as float on unconsolidated sands in the Cyclic Member, a facies interpreted as distributary mouth bar deposits in the delta-front area [5]. Dry-screening of localities led to the recovery of additional smaller shark teeth, but did not turn up additional bony fish or croc material.

The Eocene age for the fossil localities near Eames River is based upon pollen samples initially analyzed by Hopkins [16], [17], and reported by Miall [5]. Recent re-analysis of four pollen samples near Eames River (GSC samples C-26411, C-30610, C-30645, and C-30646) by Sweet [18] suggests that the localities are late early to middle Eocene in age and likely spanned the Early Eocene Climatic Optimum (EECO), based largely on overall species richness as well as abundance of *Caryapollenites* spp., *Ericipites*, *Intratriporopollenites* (Tilia), *Nyssapollenites* sp., and *Quercoidites* (oak) pollen. Presence of *Pistillipollenites* in three of the samples (absent from the coal of sample C-30646) suggests a probable minimum age of middle Eocene for the samples, while absence of *Aquilapollenites tumanganicus* Bolotnikova and closely allied species, infrequent occurrences of *Momipites* spp., and the richness of the angiosperm component of the assemblages precludes an earliest Eocene age for the samples [18].

## Materials and Methods

The fossils were collected on northern Banks Island in 2004, 2010, and 2012, and permits to conduct paleontological field research in Aulavik National Park were provided by Parks Canada, Western Arctic Field Unit. All necessary permits were obtained for the described study, which complied with all relevant regulations. The fossils from Banks Island are curated at the Canadian Museum of Nature (CMN) in Ottawa, ON, and are on loan to the University of Colorado Museum of Natural History (UCM) for study. Identifications were made based upon comparison with specimens held in collections at the UCM, the University of California Museum of Paleontology (UCMP) in Berkeley, CA, and with published descriptions and images. Terminology used to describe the crocodyliform vertebra follows Romer [19], and for gar and amiid specimens follows Grande [20] and Grande and Bemis [21], respectively. Although additional bony fish material (isolated teeth and centra) was recovered from Banks Island, it could not be referred to familial level, and therefore we did not include these non-diagnostic specimens.

## Systematic Paleontology

Actinopterygii Cope, 1887

Ginglymodi Cope, 1872

Lepisosteiformes Hay, 1929

Lepisosteidae Cuvier, 1825

*Atractosteus* Rafinesque, 1820

### 

#### Referred Specimen

**Figure 2:**
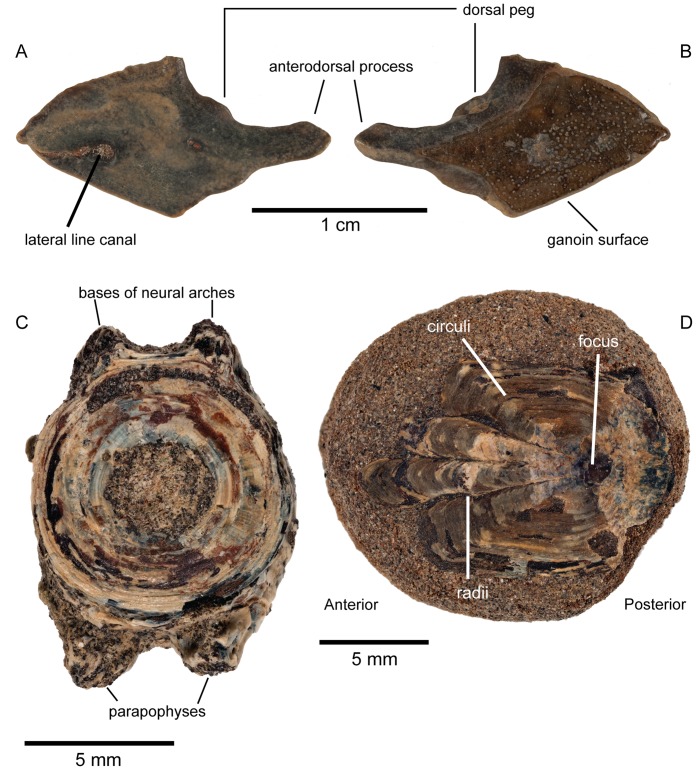
Fossils of Eocene bony fishes from northern Banks Island, NWT. CMNFV 56070, lateral line scale of *Atractosteus* from CMN Loc. BKS04-16, in medial (A) and lateral (B) views. (C) CMNFV 56069, vertebral centrum of ?Amiid. (D) CMNFV 56071, Esocid scale. C and D are from CMN Loc. BKS04-19.

CMNFV 56070, lateral line scale, Fig. 2a, b.

#### Locality and Horizon

CMN Loc. BKS04-16, N 74° 10′; W 120° 45′; Eames River, Aulavik National Park, northern Banks Island, NWT; Cyclic Member, Eureka Sound Formation (early – middle Eocene).

#### Description

CMNFV 56070 is a complete lateral line scale and readily identifiable as a gar (Fig. 2a, b). The specimen measures 18×9 mm, and has the characteristic elongate rhombic (diamond) shape of a gar scale, with a thickened bony base and an outer surface mostly covered with ganoin that bears several dozen regularly-spaced shallow perforations. The edges of the ganoin bear a relatively fine ornament of slightly serrated scalloping. The medial (inner) surface exhibits a well-formed canal, partially encased in the bony base of the scale, for carrying the mechanosensory lateral line through the scale.

The scale has a long, narrow, anteriorly-projecting anterodorsal process, which is a part of the scale that is overlapped by its neighbor, and therefore contributes to the rigidity of the interlocking scale cover in gars. The dorsal peg is, however, little more than a very low bump along the dorsal edge of the scale, just posterior to the base of the anterodrosal process. Grande [20] hypothesized that the absence of a prominent dorsal peg was a feature that may be diagnostic for gars in the genus *Atractosteus*, as opposed to its sister-genus *Lepisosteus* in which the scales typically have more prominent dorsal pegs. For this reason, as well as the close similarity in overall shape and proportions of the Banks Island scale to those of *Atractosteus* spp. illustrated by Grande [20], we assign the Banks Island scale to *Atractosteus*.

?Amiiformes Hay, 1929

?Amiidae Bonaparte, 1838

#### Referred Specimen

CMNFV 56069, vertebral centrum, Fig. 2c.

#### Locality and Horizon

CMN Loc. BKS04-19, N 74° 10′; W 120° 46′; Eames River, Aulavik National Park, northern Banks Island, NWT; Cyclic Member, Eureka Sound Formation (early – middle Eocene).

#### Description

CMNFV 56069 is a single nearly complete centrum, most likely from the mid-abdominal region and past the middle of the body, based on the relatively closely-spaced and ventrally directed parapophyses (Fig. 2c). The centrum is amphicoelous and slightly higher dorsoventrally (9.5–10 mm) than it is wide (8.5–9.0 mm), and varies from 4 to 5 mm in length, being slightly longer along its dorsal edge. The articular surfaces are rather shallowly concave, and the centrum overall has a simple, almost shark centrum-like appearance.

The bases of the neural arches are closely spaced and appear to be fused to the dorsal margin of the centrum. The larger, more prominent parapophyses ( =  the lateral components of the basiventral elements in the abdominal region, sensu Grande and Bemis [21]) are slightly more widely separated and project nearly straight ventrally, with only a hint of lateral divergence, which, as mentioned above, implies that this centrum is from a relatively posterior but still abdominal position in the vertebral column, and anterior to the caudal or ural centra.

While we cannot definitively rule out other possibilities, we note that this centrum has several features consistent with our determination that it is likely from an amiid. These include the overall proportions of the centrum (a relatively short length vs. diameter), the fact that it is perichordally ossified and amphicoelous with shallowly concave articular surfaces, the comparable shape and position of the parapophyses relative to those on posterior abdominal centra of amiids [21], the slightly pitted but otherwise relatively smooth external surface of the centrum, and the simple construction of the centrum without complexly elaborated features that are typical of centra from ‘higher’ actinopterygians. Finally, the centrum is bony, not calcified cartilage as it would be if it were from a shark, and amphicoelous, not opisthocoelous as it would be if it derived from a gar.

Teleostei sensu Patterson and Rosen 1977

Esocidae Cuvier, 1817

#### Referred Specimen

CMNFV 56071, 11 isolated teleost scales, one shown in Fig. 2d.

#### Locality and Horizon

CMN Loc. BKS04-19, N 74° 10′; W 120° 46′; Eames River, Aulavik National Park, northern Banks Island, NWT; Cyclic Member, Eureka Sound Formation (early – middle Eocene).

#### Description

Eleven isolated teleost scales were recovered from 2012 fieldwork on northern Banks Island, all preserved in small siderite concretions. The clearest and best-preserved of these is figured here (Fig. 2d) and represents Esocidae (the family that includes pikes, pickerels, and muskellunges). Scale morphological terminology follows Patterson et al. [22]. The scale is cycloid and slightly longer craniocaudally than high dorsoventrally, measuring 17 mm by 14–15 mm. The lateral surface is exposed on the siderite concretion, and exhibits very fine concentric circuli that follow the contour of the outer margin of the scale. There is variation in the appearance of the circuli, suggesting that the more distinctly visible bands may be annuli that reflect seasonal differentiation in growth and growth checks. The anterior mid-region of the scale has a demarcated anterior field portion that forms an elongate cone-shaped area set off by two distinct angled radii that reach the anterior margin of the scale; this field extends out from the center of formation (focus) of the scale, broadening towards the anterior border of the scale, and it is subdivided into two narrower regions within the anterior field by another, more medially positioned radii. The distinctive appearance of this scale very closely matches scales of extant esocids, including the Northern Pike *Esox lucius* [23].

Crocodyliformes Hay, 1930

Eusuchia Huxley, 1875

#### Referred Specimen

**Figure 3:**
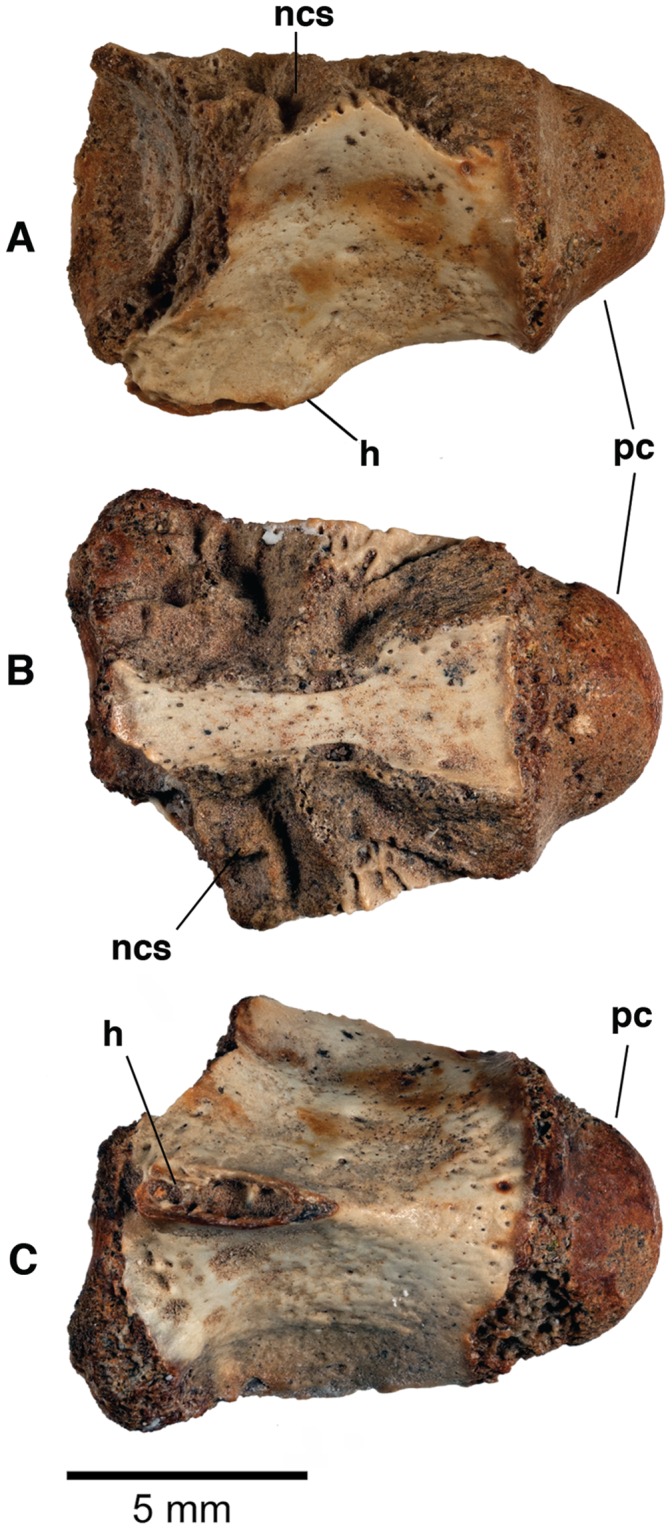
CMNFV 56059, vertebral centrum of an Eocene crocodyliform from CMN Loc. BKS04-19 on northern Banks Island, NWT. (A) Left lateral view; (B) dorsal view; (C) ventral view. h, hypapophysis; ncs, neurocentral sutural surface; pc, posterior cotyle. Scale bar equals 5 mm.

CMNFV 56059, incomplete vertebra, Fig. 3.

#### Locality and Horizon

CMN Loc. BKS04-19, N 74° 10′; W 120° 46′; Eames River, Aulavik National Park, northern Banks Island, NWT; Cyclic Member, Eureka Sound Formation (early – middle Eocene).

#### Description

CMNFV 56059 is a small procoelous vertebral centrum whose neural arch is no longer preserved (Length = 12.1 mm; Anterior width = 9.14 mm). The centrum bears a short hypapophysis on its anteroventral surface. The anterolateral surfaces are damaged on both sides, but the centrum flares anteriorly in dorsal and ventral view (Fig. 3), which indicates that parapophyses were present on the centrum adjacent to the neurocentral suture.

Because of the shortness of the hypapophysis and inferred presence of a parapophysis on the centrum, we interpret CMNFV 56059 as coming from the posterior cervical part of the vertebral column. Although not preserved, the parapophysis was adjacent, or nearly so, to the neurocentral suture. The crocodyliform parapophysis is widely separated from the neural arch in the anteriormost cervical vertebrae except for the atlas, and it gradually adopts a more dorsal position on the centrum surface until, as one approaches the cervicodorsal transition, it straddles the neurocentral suture. More anteriorly located cervical vertebrae also have longer hypapophyses.

The size of CMNFV 56059 indicates a small animal, and the vertebra compares in size with anterior dorsals on a 2-ft long *A. mississippiensis* skeleton (UCM PTC-47). In extant crocodylians, closure of the neurocentral sutures in the vertebral column follows a caudal to cranial sequence during ontogeny, with the sutures of most caudal vertebrae closed at hatching while closure of the cervicals occurs near the end of ontogeny [24]. That the neurocentral suture surface is exposed on CMNFV 56059 (Fig. 3b) indicates that the neurocentral suture was not closed. The sutural surface, however, is very rugose. Together, these indicate an animal that was immature, but possibly approaching maturity.

Procoelous vertebrae occur in several crocodyliform lineages. In most, including some putative basal eusuchians, the anterior socket is shallow and the posterior cotyle is not very prominent [25], [26], [27], [28], [29], [30]. A deep socket and prominent hemispherical cotyle is most characteristic of Crocodylia and its closest relatives [31]. Because all known non-marine crocodyliforms from the Paleogene of North America are crocodylians, we expect more complete material from this locality to put CMNFV 56059 within Crocodylia.

Although posterior cervical and anterior crocodylian dorsal vertebrae are typically not diagnostic to family, it seems probable that CMNFV 56059 belonged to an alligatorid. Most Paleogene alligatorids were small animals between 2 and 3 m in length. Alligatorids are also more cold-tolerant than other crocodylians [32], [33] and more likely to occur at high latitudes. Further, alligatorid fossils referred to *Allognathosuchus* are relatively abundant at early Eocene localities on Ellesmere Island, known from dozens of teeth and osteoderms as well as jaws and an incomplete skull, whereas no other crocodyliform taxa are known from the Arctic [3], [8].

## Discussion

Based largely on paleoclimate proxy data from the eastern and central Arctic, early – middle Eocene Arctic climate in this region has been characterized as having warm, wet summers and mild winters [9], [10], [34], [35] [36]. High-resolution carbon isotope analysis across tree rings in mummified wood from Muskox River on northern Banks Island (∼50 km south of the Eames River locality) and Stenkul Fiord on southern Ellesmere Island allow the reconstruction of seasonal precipitation patterns in the Eocene Arctic [37]. Incorporation of intra-ring δ^13^C values into a model based upon extant evergreen taxa [38] suggest that evergreen trees growing in the Eocene Arctic forests experienced three times more precipitation during summer than winter, a seasonal pattern analogous to today’s temperate forests in eastern Asia [37].

The discovery at Eames River of a fossil from an immature crocodyliform, alongside rare turtle shell fragments (too small to be identified to family), indicates that mild temperatures extended across the Arctic during early – middle Eocene time. Specifically, based upon analogy with the geographic range and climatic preferences of living crocodylians [11], the Banks Island crocodyliform infers above-freezing, year-round temperatures. This is further reinforced by the presence of gars, which are associated with mild temperate to warm conditions, and are today restricted to freshwater environments in the southeastern USA, Central America, and Cuba [20].

Given their rarity among thousands of shark teeth, the crocodyliform and turtle fossils probably were washed into the coastal delta from fully freshwater upriver habitats. While this is also a plausible explanation for the paucity of lepisosteids, amiids, and esocids, it should be noted that gars, including the extant species *Atractosteus spatula*, are known to tolerate brackish coastal environments [20], and the extant Northern Pike *Esox lucius* enters brackish coastal wetland environments in the Baltic Sea, where it is anadromous [39]. Therefore, caution is required with bony fishes in this regard because salinity and overall environmental conditions of extant taxa may not always provide an accurate guide to the tolerances of their relatives in the fossil record.

Previous work from the Eocene Canadian Arctic, summarized in Eberle and Greenwood [8] and Estes and Hutchison [3], identified gars (cf. *Lepisosteus* sp.), amiids (*Cyclurus fragosa* and *Amia* cf. *A. pattersoni*), and esocids (cf. *Esox* sp.) on Ellesmere, which means that, at least to the family level, the low-diversity bony fish faunas of Ellesmere and Banks islands, separated by some 40 degrees of longitude and over 1,000 km, are essentially identical. This suggests a pan-High Arctic bony fish fauna of relatively basal groups that extended around the margin of the Eocene Arctic Ocean, inhabiting freshwater and perhaps low salinity marginal marine settings during the EECO. This family-level biogeographic hypothesis could be further refined if additional fish specimens are recovered that are identifiable to a finer taxonomic level, but at present our suggestion of a low-diversity, pan-High Arctic pattern in bony fishes is consistent with our current understanding of the Banks Island and comparable Arctic Eocene faunas. Along with the Banks and Ellesmere island occurrences, there is a relevant High Arctic fossil amiid record from the Svalbard Archipelago – *Pseudamiatus heintzi* (Lehman, 1951) [40], a partially articulated specimen collected at 78°N on the west coast of Spitsbergen. The specimen was recovered from a similar deltaic depositional environment as the Banks material, so it is uncertain whether the fish inhabited a marine, brackish, or freshwater environment. *Pseudamiatus* was first described as Eocene [40], but more recently the Firkanten Formation from which it derives has been re-interpreted as lower Paleocene (Danian) [41], [21].

The survivors, in an Arctic context, of this Eocene High Arctic grouping are the esocids, today represented by the Northern Pike, *Esox lucius*, which is circumglobal in Holarctic freshwater environments as high as 74° N [42]. In contrast, the ranges of gars and bowfins have retracted since the Eocene into their present-day lower-latitude and environmentally more mild distributions.

Arctic crocodylians could resolve several longstanding biogeographic questions, including the biogeographic origins of Asian alligatorids. There are two living species of *Alligator* – one in North America (the American alligator, *Alligator mississippiensis*) and another in China (the critically endangered Chinese alligator, *A. sinensis*). Because alligators are intolerant of salt water [43], a non-marine dispersal corridor, such as Beringia, probably explains the presence of an otherwise North American clade in eastern Asia [44], [45], [46]. Fossil evidence puts the minimum divergence time between the two lineages in the early Miocene [47], but high-latitude dispersal routes would not have been within crocodylian thermal preferences at that time [11]. Molecular data generally put the *mississippiensis-sinensis* split in the Paleogene [48], [49], [50], when climatic conditions were more favorable for high-latitude alligatorids, and presence of a crocodyliform (and probable alligatorid) in Canada’s western Arctic is consistent with this hypothesis.

A longstanding biogeographic question regards the origin of Asian alligatorids. Phylogenetic analyses thus far have rejected a close relationship between Paleogene alligatorids and either living species of *Alligator* [44], [45], [46], [51], but if the *mississippiensis-sinensis* split occurred in the Paleogene and followed a Beringian route to Asia, we would predict the discovery of Arctic fossils within crown *Alligator*. Further field research in the region may uncover more diagnostic material that can resolve this biogeographic question.
